# Reduced and Nonreduced Genomes in *Paraburkholderia* Symbionts of Social Amoebas

**DOI:** 10.1128/msystems.00562-22

**Published:** 2022-09-13

**Authors:** Suegene Noh, Benjamin J. Capodanno, Songtao Xu, Marisa C. Hamilton, Joan E. Strassmann, David C. Queller

**Affiliations:** a Department of Biology, Colby College, Waterville, Maine, USA; b Brotman Baty Institute for Precision Medicine, Seattle, Washington, USA; c University Program in Genetics and Genomics, Duke University, Durham, North Carolina, USA; d Department of Biology, Washington University in St. Louis, St. Louis, Missouri, USA; Univ. California Merced

**Keywords:** symbiosis, protist, *Burkholderia*, *Dictyostelium*, genome reduction

## Abstract

The social amoeba Dictyostelium discoideum is a predatory soil protist frequently used for studying host-pathogen interactions. A subset of D. discoideum strains isolated from soil persistently carry symbiotic *Paraburkholderia*, recently formally described as *P. agricolaris*, *P. bonniea*, and *P. hayleyella*. The three facultative symbiont species of D. discoideum present a unique opportunity to study a naturally occurring symbiosis in a laboratory model protist. There is a large difference in genome size between *P. agricolaris* (8.7 million base pairs [Mbp]) versus *P. hayleyella* and *P. bonniea* (4.1 Mbp). We took a comparative genomics approach and compared the three genomes of D. discoideum symbionts to 12 additional *Paraburkholderia* genomes to test for genome evolution patterns that frequently accompany host adaptation. Overall, *P. agricolaris* is difficult to distinguish from other *Paraburkholderia* based on its genome size and content, but the reduced genomes of *P. bonniea* and *P. hayleyella* display characteristics indicative of genome streamlining rather than deterioration during adaptation to their protist hosts. In addition, D. discoideum-symbiont genomes have increased secretion system and motility genes that may mediate interactions with their host. Specifically, adjacent BurBor-like type 3 and T6SS-5-like type 6 secretion system operons shared among all three D. discoideum-symbiont genomes may be important for host interaction. Horizontal transfer of these secretion system operons within the amoeba host environment may have contributed to the unique ability of these symbionts to establish and maintain a symbiotic relationship with D. discoideum.

**IMPORTANCE** Protists are a diverse group of typically single cell eukaryotes. Bacteria and archaea that form long-term symbiotic relationships with protists may evolve in additional ways than those in relationships with multicellular eukaryotes such as plants, animals, or fungi. Social amoebas are a predatory soil protist sometimes found with symbiotic bacteria living inside their cells. They present a unique opportunity to explore a naturally occurring symbiosis in a protist frequently used for studying host-pathogen interactions. We show that one amoeba-symbiont species is similar to other related bacteria in genome size and content, while the two reduced-genome-symbiont species show characteristics of genome streamlining rather than deterioration during adaptation to their host. We also identify sets of genes present in all three amoeba-symbiont genomes that are potentially used for host-symbiont interactions. Because the amoeba symbionts are distantly related, the amoeba host environment may be where these genes were shared among symbionts.

## INTRODUCTION

The social amoeba Dictyostelium discoideum (*Eumycetozoa*; *Dictyosteliales*) is a predatory soil protist frequently used for studying host-pathogen interactions (1–3). It is also an emerging model for host-microbe symbiosis in the broad sense, which we define here as an intimate association between a eukaryotic host and a prokaryote symbiont that can result in positive, neutral, or negative fitness consequences in both parties involved (4–6). A subset of D. discoideum strains isolated from soil persistently carry intracellular Gram-negative *Paraburkholderia* (*Betaproteobacteria*; *Burkholderiales*) (7–9). Multilocus sequence typing analyses and whole-genome phylogenies showed that these symbionts comprise two independent clades (9, 10). Subsequently, *P. agricolaris* and the two sister species *P. bonniea* and *P. hayleyella* were formally described as new species sufficiently different from any other previously described *Paraburkholderia* using genetic and phenotypic evidence (10).

The three *Paraburkholderia* symbionts of D. discoideum present a unique opportunity to study a naturally occurring symbiosis in a laboratory model protist. They additionally present opportunities for insight into the diversity of protist-prokaryote symbioses, which are understudied compared to the symbiotic relationships of multicellular eukaryotes and their microbial symbionts (11). The association between D. discoideum and its *Paraburkholderia* symbionts appears to be facultative, and these symbionts are able to simultaneously maintain a free-living and host-associated lifestyle (8, 9). With *Paraburkholderia* the fitness outcomes to host and symbiont appear to be context dependent (12), as with most facultative host-microbe symbioses (6). D. discoideum amoeba hosts generally suffer negative fitness consequences of association. When amoebas are infected with their *Paraburkholderia* symbionts in the lab, the hosts tend to eat less food bacteria during vegetative growth, migrate shorter distances as slugs during their multicellular social cycle, form shorter and smaller volume fruiting bodies, produce fewer spores, and carry other bacteria alive (secondary carriage) into their next vegetative growth cycle (7, 8, 13, 14). However, potentially important context-dependent fitness benefits to the host may be (i) increased availability of food bacteria in relatively inhospitable environments as a result of secondary carriage, and (ii) improved competitive ability against other D. discoideum strains by potentially passing on symbiont infections or releasing *Paraburkholderia* secretions in a defensive manner (7, 15). We know less about fitness outcomes of association for *Paraburkholderia* symbionts, but *P. hayleyella* (though not *P. agricolaris*) reaches higher population densities in the presence of D. discoideum compared to on its own in soil medium (16). While not a direct demonstration of any fitness benefits, *P. agricolaris* and *P. hayleyella* show positive chemotaxis toward D. discoideum supernatant (17). Later, D. discoideum was discovered to also associate with unculturable Chlamydiae and *Amoebophilus* symbionts but with no obvious fitness effects of infection or clear patterns of coinfection with *Paraburkholderia* (18).

We present a comparative genomics analysis of the three types of strains of *Paraburkholderia* isolated from D. discoideum (*P. agricolaris* BaQS159, *P. hayleyella* BhQS11, and *P. bonniea* BbQS859). We isolated all three strains from D. discoideum hosts collected at Mountain Lake Biological Station in Virginia, USA. Notably, there is a large difference in genome size between *P. agricolaris* (8.7 million base pairs [Mbp]) versus *P. hayleyella* and *P. bonniea* (4.1 Mbp) and in GC content (62% versus 59%) (9, 10). Genome reduction is a pattern associated with long-term host association in many symbiotic bacteria (19–25), including pathogenic *Burkholderia* (26) and amoeba-associated *Legionella* (27, 28). Therefore, we investigated any significant differences in genome characteristics in the genomes of D. discoideum symbionts and particularly in the reduced genomes of *P. hayleyella* and *P. bonniea*. Based on what we know from the best-studied endosymbiont genomes of multicellular animals, we looked for patterns that frequently accompany host adaptation, such as fewer genes in functional categories related to metabolism, DNA repair, and gene regulation (29, 30).

Because the ability to infect D. discoideum appears to be a shared derived trait among *Paraburkholderia* symbionts of D. discoideum, we focused several analyses on shared orthologous genes across the three genomes in comparison with other *Paraburkholderia*. Given the estimated large phylogenetic distance between the two D. discoideum-symbiont clades (9, 10), we paid particular attention to shared horizontally transferred genetic elements. Horizontal gene transfer generally contributes to an increase in prokaryote genomic repertoires but is subject to evolutionary processes, including selection and drift as with the rest of the genome (31–34). In the context of symbiosis, key horizontally transferred genes can enable new symbiotic relationships (e.g., symbiosis islands) (35). If host adaptation-induced genome reduction is ongoing, we expected symbiont genomes to show signs of instability in the form of excess nonfunctional horizontally transferred genetic elements (e.g., insertion sequence [IS] elements or pseudogenes) (36). IS elements in particular connect the themes of genome reduction and horizontally transferred genetic elements. They often proliferate during earlier stages of host adaptation and enable genome rearrangements and deterioration (37, 38), eventually leading to the highly reduced genomes seen in obligate symbionts.

## RESULTS

### D. discoideum symbionts represent two distinct categories, reduced versus nonreduced size genomes.

Sequencing using PacBio technology indicated that the genomes of all three D. discoideum*-*symbiont species are each comprised of two chromosomes, albeit resulting in different total genome sizes. The *P. agricolaris* genome was more than twice the size of both *P. bonniea* and *P. hayleyella* (8.7 versus 4.1 million basepairs). The overall gene content (CDS) comparison was also proportionate, with approximately 7,700 genes predicted for *P. agricolaris* as opposed to approximately 3,600 genes for the reduced genomes (Table 1). The genome size and gene count of *P. agricolaris* are on par with other the *Paraburkholderia* genomes we examined (Table 2).

**TABLE 1:** Genome statistics of *Paraburkholderia* symbionts of D. discoideum

Genome statistics	*P. agricolaris* BaQS159	*P. bonniea* BbQS859	*P. hayleyella* BhQS11
Scaffold count	2	2	2
Genome size (chr1; chr2)	8,721,420 (4,816,966; 3,904,454)	4,098,182 (3,175,376; 922,806)	4,125,700 (3,295,139; 830,561)
GC content (%)	61.6	58.7	59.2
Genes (total)	7,811	3,600	3,686
CDS (total)	7,721	3,531	3,610
Pseudogene count	579	265	315
rRNA count	18	12	12
tRNA count	71	56	63
Locality (host)	D. discoideum in Virginia, USA	D. discoideum in Virginia, USA	D. discoideum in Virginia, USA

**TABLE 2:** Genome statistics of other representative *Paraburkholderia* strains for comparison

Genome statistics	*P. fungorum* ATCC BAA-463	*P. sprentiae* WSM5005	*P. terrae DSM* 17804	*P. xenovorans* LB400
Scaffold count	4	5	4	3
Genome size (total)	9,058,983	7,829,542	10,062,489	9,702,951
GC content	61.8	63.2	61.9	62.6
Genes (total)	8,260	7,185	9,045	8,760
CDS (total)	8,174	7,087	8,957	8,675
Pseudogene count	746	857	803	845
rRNA count	18	21	18	18
tRNA count	67	76	69	66
Locality (host)	White-rot fungus Phanerochaete chrysosporium in Sweden	Domesticated legume *Lebeckia ambigua* in Australia	Broad-leaved forest soil in South Korea	Polychlorinated biphenyl (PCB)-contaminated soil in USA

**FIG 1:**
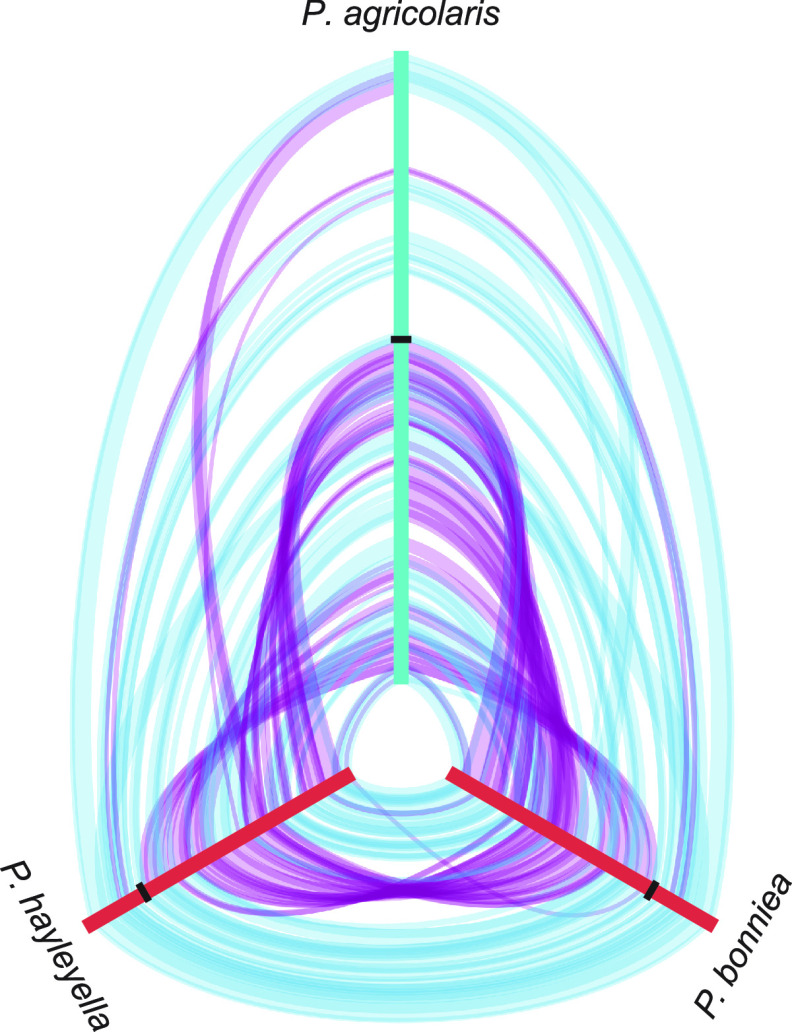
Hive plot of whole-genome comparisons of D. discoideum-symbiont genomes. Locally colinear blocks between pairs of genomes are shown as bands that connect the axes (genomes). Only blocks above the median size are shown. Alignment of locally colinear blocks are distinguished between forward (blue) and reverse (purple) orientation. Axes are oriented center out, and boundaries between chromosomes are shown as ticks.

Whole-genome alignments of all 10 finished genomes found 153 locally colinear blocks, ranging in sizes as small as 262 bp and as large as 208,252 bp in the *P. agricolaris* genome. *P. bonniea* and *P. hayleyella* share with each other considerable synteny but also possess a large inverted region relative to each other (Fig. 1). Both of these reduced genomes show extensive genome rearrangement compared to the genome of *P. agricolaris*, or any of the other *Paraburkholderia* genomes (Fig. 1 and Fig. S1 in the supplemental material). The genome of *P. agricolaris* shares a large degree of synteny with other *Paraburkholderia* genomes in chromosome 1 as indicated by the overall lack of gaps toward the center of each hive plot (Fig. S1).

10.1128/msystems.00562-22.6 FIG S1 Hive plots of D. discoideum-symbiont genomes compared to four other *Paraburkholderia* genomes *P. agricolaris* (a), *P. bonniea* (b), and *P. hayleyella* (c). Locally colinear blocks between pairs of genomes are shown as bands that connect the axes (genomes). Only blocks above the median size are shown for visual clarity. Alignment of locally colinear blocks are distinguished between forward (blue) and reverse (purple) orientation. Axes are oriented center out, and boundaries between chromosomes are shown as ticks. The two reduced genomes show considerable genome rearrangement while chromosome 1 is largely similar for these *Paraburkholderia* genomes as indicated by the overall lack of gaps toward the center of each hive plot. The origin of chromosome 1 appears to be slightly different for other *Paraburkholderia* genomes (see connections between the center origin and regions close to the tick mark indicating the boundary between linearized chromosomes 1 and 2). Download FIG S1, PDF file, 1.1 MB. Copyright © 2022 Noh et al. 2022 Noh et al. https://creativecommons.org/licenses/by/4.0/ This content is distributed under the terms of the Creative Commons Attribution 4.0 International license.

We found few IS elements in the D. discoideum-symbiont genomes. *P. agricolaris* has the IS elements IS1090 (6 copies) and ISBmu21 (1 copy), *P. bonniea* has ISBp1 (2 copies) and ISBuph1 (3 copies), and *P. hayleyella* has a single ISPa37 in their genomes (Table S2). Among the other *Paraburkholderia* genomes we examined, the highest number of IS elements was found in *P. xenovorans* LB400 (62 total), while others possessed intermediate numbers ranging from 5 in *P. sprentiae* to 21 in *P. fungorum*. For reference, genomes of B. mallei possess between 166 and 218 IS elements, many of which were flanking regions that were randomly lost among the examined strains in what appears to be ongoing genome reduction (37). There was also no evidence of excess pseudogenes in the reduced genomes relative to other *Paraburkholderia* genomes (Table 1). Double-strand break repair pathways (KEGG map03440) were complete in all three D. discoideum-symbiont genomes. As genomes with ongoing genome reduction often have numerous IS elements and pseudogenes, and incomplete double-strand break repair pathways, the combined evidence suggests that all three D. discoideum-symbiont genomes are relatively stable and the two reduced genomes are currently not in flux.

10.1128/msystems.00562-22.2 TABLE S2 Insertion sequence (IS) elements found in D. discoideum-symbiont *Paraburkholderia* genomes. Download Table S2, DOCX file, 0.01 MB. Copyright © 2022 Noh et al. 2022 Noh et al. https://creativecommons.org/licenses/by/4.0/ This content is distributed under the terms of the Creative Commons Attribution 4.0 International license.

### The reduced genomes of D. discoideum symbionts show evidence of functional adaptation to the host environment.

**FIG 2:**
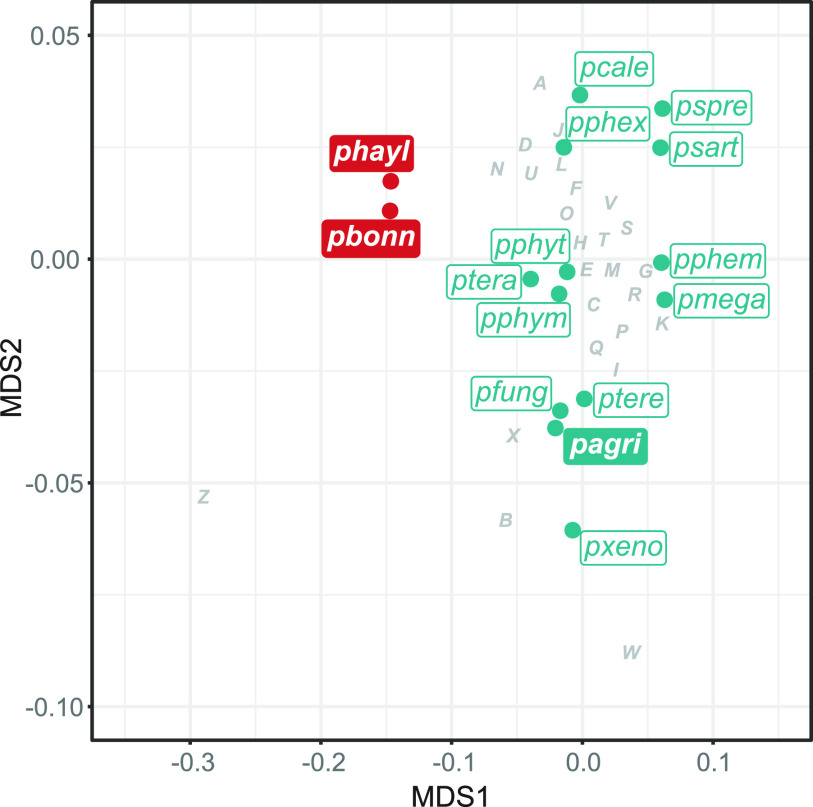
Comparison of reduced (red) and nonreduced (turquoise) genomes in terms of their functional compositions in nonmetric multidimensional space. The contributions of COG categories are projected with minor adjustments to avoid overlap with other features (pagri, *P. agricolaris*; pbonn, *P. bonniea*; phayl, *P. hayleyella*; pcale, *P. caledonica*; pfung, *P. fungorum*, pmega, *P. megapolitana*, pphem, *P. phenazinium*; pphex, *P. phenoliruptrix*; pphym, *P. phymatum*; pphyt, *P. phytofirmans*; psart, *P. sartisoli*; pspre, *P. sprentiae*; ptera, *P. terricola*; ptere, *P. terrae*; pxeno, *P. xenovorans*) (COG categories: J, Translation, ribosomal structure and biogenesis; A, RNA processing and modification; K, Transcription; L, Replication, recombination and repair; B, Chromatin structure and dynamics; D, Cell cycle control, cell division, chromosome partitioning; Y, Nuclear structure; V, Defense mechanisms; T, Signal transduction mechanisms; M, Cell wall/membrane/envelope biogenesis; N, Cell motility; Z, Cytoskeleton; W, Extracellular structures; U, Intracellular trafficking, secretion, and vesicular transport O, Posttranslational modification, protein turnover, chaperones; X, Mobilome: prophages, transposons; C, Energy production and conversion; G, Carbohydrate transport and metabolism; E, Amino acid transport and metabolism; F, Nucleotide transport and metabolism; H, Coenzyme transport and metabolism; I, Lipid transport and metabolism; P, Inorganic ion transport and metabolism; Q, Secondary metabolites biosynthesis, transport and catabolism; R, General function prediction only; S, Function unknown.

For each D. discoideum-symbiont genome, 65 to 68% of genes were annotated with Clusters of Orthologous Groups (COG) (Fig. S2), and 53 to 61% with Kyoto Encyclopedia of Genes and Genomes (KEGG) Orthology (KO). The agglomerative clustering and nonmetric multidimensional scaling (NMDS) analyses of COG category representation across genomes resulted in *P. bonniea* and *P. hayleyella* clustering with each other and apart from other *Paraburkholderia*, including *P. agricolaris* (Fig. 2). Further investigation of specific functional differences between the two groups (reduced genomes versus nonreduced) indicated nine COG categories that were significantly different (exactTest, false discovery rate ≪ 0.01). Of these, four were consistently different in both normalized and raw counts (Fig. S3): fewer genes were detected in the reduced genomes of *P. bonniea* and *P. hayleyella* for Transcription (category K), Carbohydrate transport and metabolism (G), and Inorganic ion transport and metabolism (P), and more genes were found in the reduced genomes for Cell motility (N).

10.1128/msystems.00562-22.7 FIG S2 (a) Composition of each D. discoideum-symbiont genome divided into core genes shared among all *Paraburkholderia* examined, “dicty” genes shared among all three D. discoideum-symbiont genomes but excluding core genes, and all other accessory genes. (b) Compositions of *P. agricolaris* and 4 representative *Paraburkholderia* genomes divided into core genes shared among all *Paraburkholderia* examined, “dicty” genes shared among all three D. discoideum-symbiont genomes but excluding core genes, and all other accessory genes. COG categories: J, Translation, ribosomal structure and biogenesis; A, RNA processing and modification; K, Transcription; L, Replication, recombination and repair; B, Chromatin structure and dynamics; D, Cell cycle control, cell division, chromosome partitioning; Y, Nuclear structure; V, Defense mechanisms; T, Signal transduction mechanisms; M, Cell wall/membrane/envelope biogenesis; N, Cell motility; Z, Cytoskeleton; W, Extracellular structures; U, Intracellular trafficking, secretion, and vesicular transport O, Posttranslational modification, protein turnover, chaperones; X, Mobilome: prophages, transposons; C, Energy production and conversion; G, Carbohydrate transport and metabolism; E, Amino acid transport and metabolism; F, Nucleotide transport and metabolism; H, Coenzyme transport and metabolism; I, Lipid transport and metabolism; P, Inorganic ion transport and metabolism; Q, Secondary metabolites biosynthesis, transport and catabolism; R, General function prediction only; S, Function unknown. Download FIG S2, EPS file, 2.1 MB. Copyright © 2022 Noh et al. 2022 Noh et al. https://creativecommons.org/licenses/by/4.0/ This content is distributed under the terms of the Creative Commons Attribution 4.0 International license.

10.1128/msystems.00562-22.8 FIG S3 Comparison of significantly different COG functional categories between reduced and nonreduced *Paraburkholderia* genomes. Normalized (a) and raw (b) gene counts are shown. Categories K, N, G, and P were consistently different in the same direction for reduced genomes versus nonreduced genomes. Download FIG S3, EPS file, 1.9 MB. Copyright © 2022 Noh et al. 2022 Noh et al. https://creativecommons.org/licenses/by/4.0/ This content is distributed under the terms of the Creative Commons Attribution 4.0 International license.

**FIG 3:**
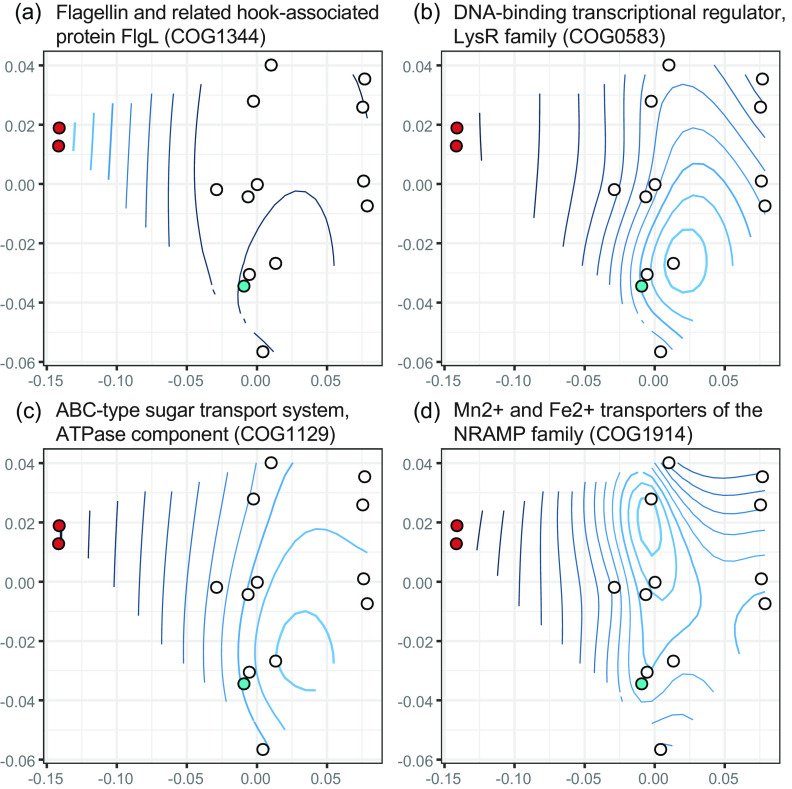
Representative individual COGs belonging to categories Cell motility (a), Transcription (b), Carbohydrate transport and metabolism (c), and Inorganic ion transport and metabolism (d) that were significantly overrepresented (a) or underrepresented (b–d) in the reduced genomes of D. discoideum symbionts. Contours of abundances are superimposed on the nonmetric multidimensional space from Fig. 2. *P. bonniea* and *P. hayleyella* are shown as red points to the left, while *P. agricolaris* is distinguished from the other genomes (white) as a turquoise point. Lighter blue contour lines indicate higher abundance compared to darker blue lines.

We looked within each COG category that was significantly different between reduced and nonreduced genomes in more detail. First, we found several flagella biosynthesis, basal body, and hook protein COGs that were more abundant in the reduced genomes than expected (Fig. 3a). Flagella are often associated with bacterial virulence, not only through providing motility but also adhesion, invasion, and the secretion and regulation of virulence factors (39, 40). Among *Burkholderia*, B. pseudomallei flagella have been shown to be necessary for postinvasion virulence in mice (41). B. pseudomallei and B. thailandensis each have two flagellar clusters, and in B. thailandensis the second cryptic cluster is involved in postinvasion intracellular motility (42). We found two flagellar clusters in *P. bonniea* compared to one in the other D. discoideum-symbiont genomes (see also “Shared secretion systems may mediate D. discoideum - Paraburkholderia-symbiont interactions”).

The other significant COG categories were less abundant in the reduced genomes than expected (Fig. 3b to d). Many families of transcriptional regulator COGs were less abundant in the reduced genomes, as is often seen with reduced symbiotic bacterial genomes (23, 43). Similarly, several ATP binding cassette (ABC)-type sugar and metal ion transporter COGs were less abundant in the reduced genomes. ABC transporters are often reduced in number in bacteria with intracellular niches compared to extracellular or environmental ones, as intracellular environments are relatively more stable compared to extracellular environments (44, 45).

Analysis with KEGG mapper reconstruction confirmed several missing sugar transport systems in the reduced genomes compared to *P. agricolaris*, including sorbitol/mannitol, l-arabinose, galactofuranose, d-xylose, fructose, and rhamnose. Genes encoding iron (III) transporters were also absent in the two reduced symbiont genomes compared to nonreduced *P. agricolaris*. A similar analysis of ABC transporters also revealed the presence of heme exporter proteins in both reduced genomes but not in *P. agricolaris* and a capsular polysaccharide transport system in *P. bonniea* only. We also examined two-component system (TCS) transporters with KEGG mapper because pathogenic *Burkholderia* have multiple two-component systems related to virulence in plant and animal infection models (46). Compared to *P. agricolaris*, the reduced genomes lacked genes encoding nitrate reductase proteins and chemotaxis proteins typically involved in biofilm formation through cyclic di-GMP regulation. In free-living B. pseudomallei, these two two-component systems are linked, as the presence of nitrate has been shown to reduce intracellular cyclic di-GMP levels and inhibit biofilm formation (47). It appears these two-component systems and the aforementioned transporters have not been maintained under selection during host adaptation and genome reduction in *P. bonniea* and *P. hayleyella*.

### The reduced D. discoideum-symbiont genomes may experience a combination of stronger and relaxed purifying selection relative to other Paraburkholderia genomes.

**FIG 4:**
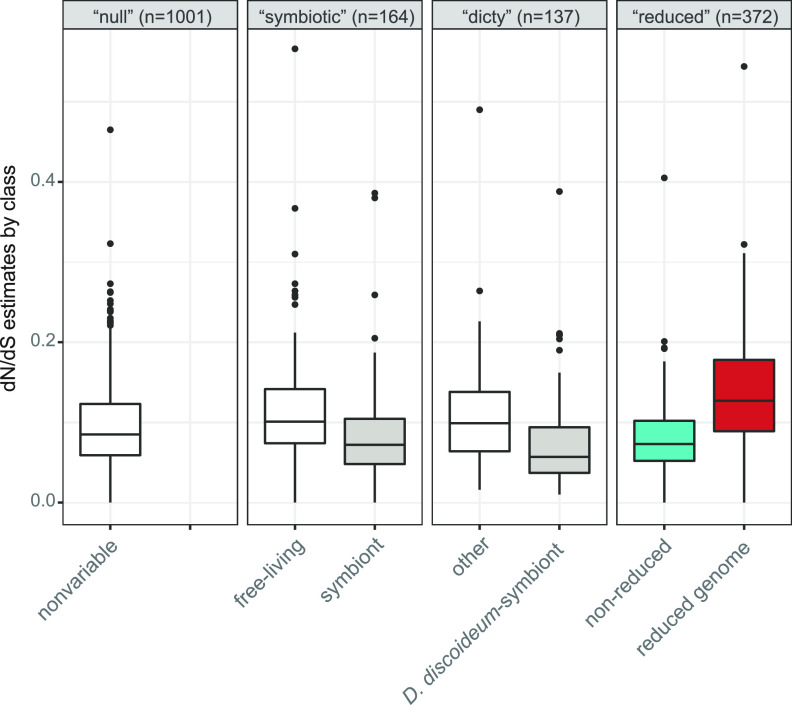
Core genes divided into the hypothesis that best predicts their patterns of molecular evolution. Core genes included genes evolving under stronger selective constraints with significantly lower dN/dS in genomes of symbionts of D. discoideum or other eukaryotes (“symbiotic” and “dicty”) and genes showing evidence of relaxed selective constraints with significantly higher dN/dS in the reduced genomes of *P. bonniea* and *P. hayleyella* (“reduced”). *P. bonniea* and *P. hayleyella* genes are included in the groups: symbiont, D. discoideum-symbiont, and reduced genome within each hypothesis.

We identified 1,673 core genes shared by the 15 *Paraburkholderia* species genomes we investigated (Fig. S2). When we examined dN/dS as a signature of molecular evolution, the majority of the *Paraburkholderia* core genes showed nonsignificant variation in selection pressure across the species phylogeny. However, a large proportion of core genes (~40%) showed an alternative pattern of molecular evolution (Table 3). These genes show one of two patterns of molecular evolution: those that appear to experience increased selection pressure and significantly lower dN/dS once symbiotically associated with eukaryotes or specifically with D. discoideum (“symbiotic” and “dicty”; both Wilcoxon test *P* ≪ 0.01), and those that show evidence of relaxed selection and significantly higher dN/dS in genomes of reduced size (“reduced”; Wilcoxon test *P* ≪ 0.01) (Fig. 4). These results indicate that the reduced genomes of *P. bonniea* and *P. hayleyella* possess a combination of genes experiencing stronger selective constraints and genes under weaker selective constraints relative to the genomes of other *Paraburkholderia*. This pattern is in contrast to genomes of obligate symbionts where the majority of genes are experiencing genetic drift and weaker selection constraints across their entire genomes (48, 49).

**TABLE 3:** Hypotheses tested regarding molecular evolution in the 1,673 core genes shared across 15 *Paraburkholderia* genomes

Hypothesis	No. of core genes	Detailed description
“null”	1001	Selection pressure does not vary across the tree
“symbiotic”	163	Selection pressure is different when species are free-living versus symbiotically associated with a eukaryotic host (2 rate ratios)
“dicty”	137	Selection pressure is different when species are unassociated versus symbiotically associated with D. discoideum (2 rate ratios)
“reduced”	372	Selection pressure is different in species with reduced genomes (*P. bonniea and P. hayleyella*) (2 rate ratios)

The three D. discoideum-symbiont genomes shared 1,977 genes total, including the 1,673 core genes (Fig. S2). Of the 1977 D. discoideum-symbiont-shared genes (inclusive of core genes), 120 were not orthologous to genes found in any of the other *Paraburkholderia* genomes we compared. These genes included type 3 and type 6 secretion system component genes (see “Shared secretion systems may mediate D. discoideum - Paraburkholderia-symbiont interactions”), *bhuRSTUV* genes, and helix-turn-helix motif-containing GntR and LysR transcriptional regulators. *Burkholderia* heme uptake (*bhu*) genes are thought to be important for intracellular iron acquisition in B. pseudomallei (50) and are related to *Bordatella* heme utilization (*bhu*) genes that are virulence factors in mammalian and avian host infection (51, 52). Transcriptional regulators with helix-turn-helix motifs have been frequently associated with virulence in pathogens (53).

### The relationship between D. discoideum and its symbionts is unlikely to be based on amino acid exchange.

The gradual loss of essential amino acid biosynthetic ability is a feature of genome reduction in many microbial symbionts that have nutrient exchange relationships with their hosts (54–56). However, nutrient-dependent relationships are less likely in protist-prokaryote symbioses because protist host diets tend to be much more diverse compared to multicellular eukaryotes (11). Accordingly, the three D. discoideum-symbiont species are predicted to synthesize all essential amino acids (Table S3), albeit with some variation in degrees of confidence. High-confidence candidates were identified for each of the steps of amino acid biosynthesis in *P. agricolaris*, but some pathways included medium confidence steps in the other two species with reduced genomes. In *P. bonniea*, the l-arginine biosynthesis pathway contained one medium confidence enzyme (Ornithine carbamoyltransferase *argI*) that was a lower coverage match (78%) than the high-confidence threshold (>80%). There is more evidence for a potential breakdown of essential amino acid synthesis in *P. hayleyella*. *P. hayleyella* had four potential gaps in its amino acid biosynthesis pathways. The l-isoleucine, l-leucine, and l-valine pathways shared a single medium-confidence enzyme candidate that is potentially a l-arabonate dehydratase rather than the necessary dihydroxy-acid dehydratase *ilvD* based on ublast bit scores. The l-tryptophan pathway had two medium confidence enzyme candidates for phosphoribosylanthranilate isomerase (*PRAI*), and the better scoring one was a lower coverage match (71%) than the high-confidence threshold. However, given the degree of genome reduction that has already occurred in the reduced-genome D. discoideum symbionts, we consider it unlikely that the symbiotic relationship is based on amino acid exchange as essential amino acid synthesis pathways appear largely intact.

10.1128/msystems.00562-22.3 TABLE S3 Amino acid biosynthesis pathways predicted in D. discoideum-symbiont *Paraburkholderia* genomes. Download Table S3, DOCX file, 0.03 MB. Copyright © 2022 Noh et al. 2022 Noh et al. https://creativecommons.org/licenses/by/4.0/ This content is distributed under the terms of the Creative Commons Attribution 4.0 International license.

### D. discoideum-symbiont genomes share few recently horizontally transferred genetic elements.

We looked for evidence of shared horizontally transmitted genetic elements. We identified 38, 29, and 27 genomic islands in each D. discoideum-symbiont genome (*P. agricolaris*, *P. bonniea*, and *P. hayleyella*), but none of the predicted genomic islands were closely related to a genomic island in another D. discoideum-symbiont genome. We found 133, 109, and 120 individually horizontally transferred genes in each D. discoideum-symbiont genome. One candidate was shared among all three genomes (type VI secretion system contractile sheath, large subunit) while two additional candidates were shared by *P. bonniea* and P*. hayleyella* (PIN family putative toxin-antitoxin system, toxin component; class I SAM-dependent methyltransferase). The scarcity of easily identified shared horizontally transferred genetic elements suggests it is unlikely that a recent horizontal gene transfer event substantially contributed to the shared ability of these symbionts to persistently infect D. discoideum. If such an event had occurred, any such genes seem to have experienced amelioration over evolutionary time and cannot easily be distinguished from the rest of the genome (57).

### Shared secretion systems may mediate D. discoideum* - Paraburkholderia*-symbiont interactions.

**FIG 5:**
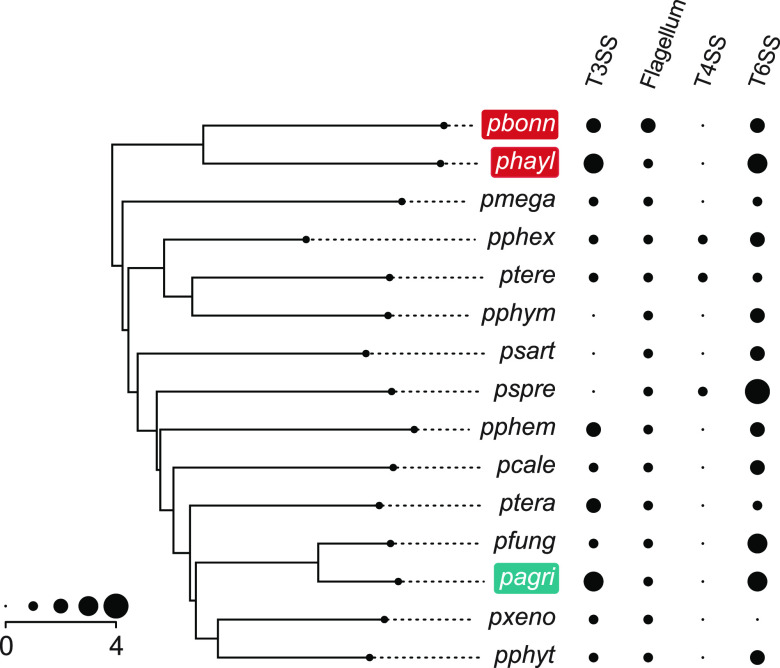
The abundances of secretion systems detected in D. discoideum-symbiont genomes and other *Paraburkholderia*. For the type 4 secretion system, only protein secretion (as opposed to conjugation-related) T4SS abundances are shown. The phylogeny is a species tree of the 15 *Paraburkholderia* genomes we examined, reduced from the larger species tree in Brock et al. (10).

**FIG 6:**
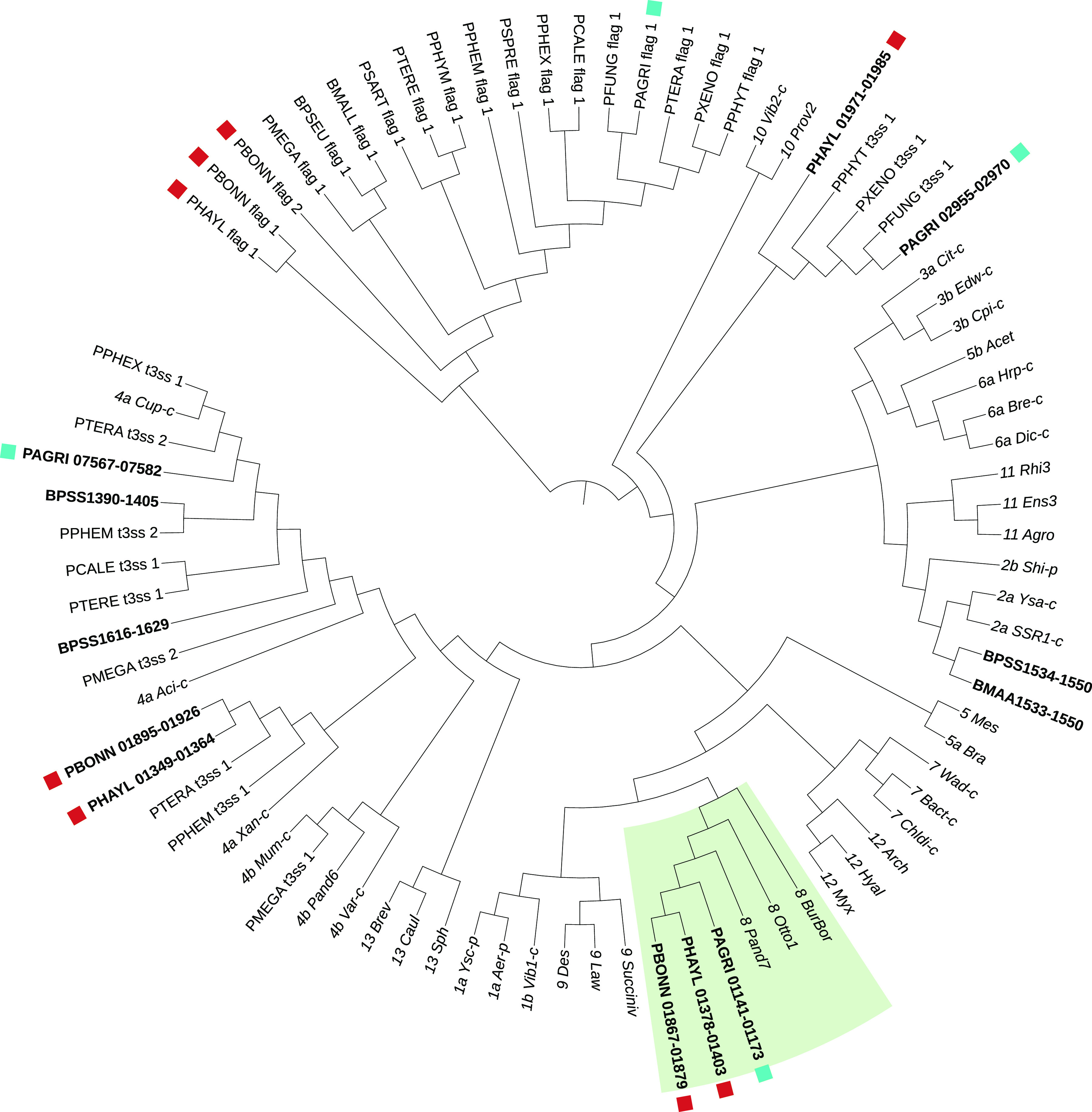
Type 3 secretion systems and flagella categorized using the conserved component genes sctJ (inner membrane ring; IPR003282), sctN (ATPase; IPR005714), and sctV (export apparatus; IPR006302). Branch lengths were ignored to improve readability of the ASTRAL tree topology. T3SS categories precede the name of the operon (e.g., “*8 Pand7*” is operon Pand7 belonging to category 8) downloaded from T3Enc database v1.0 (Hu et al. [135]). Tip labels for T3SS in the three D. discoideum-symbiont genomes, B. mallei (BMAA), or B. pseudomallei (BPSS) are shown in bold font face with gene IDs for ease of cross-reference. D. discoideum-symbiont genome T3SS operons are marked with a square symbol, and the clade containing the shared T3SS operon is shaded.

**FIG 7:**
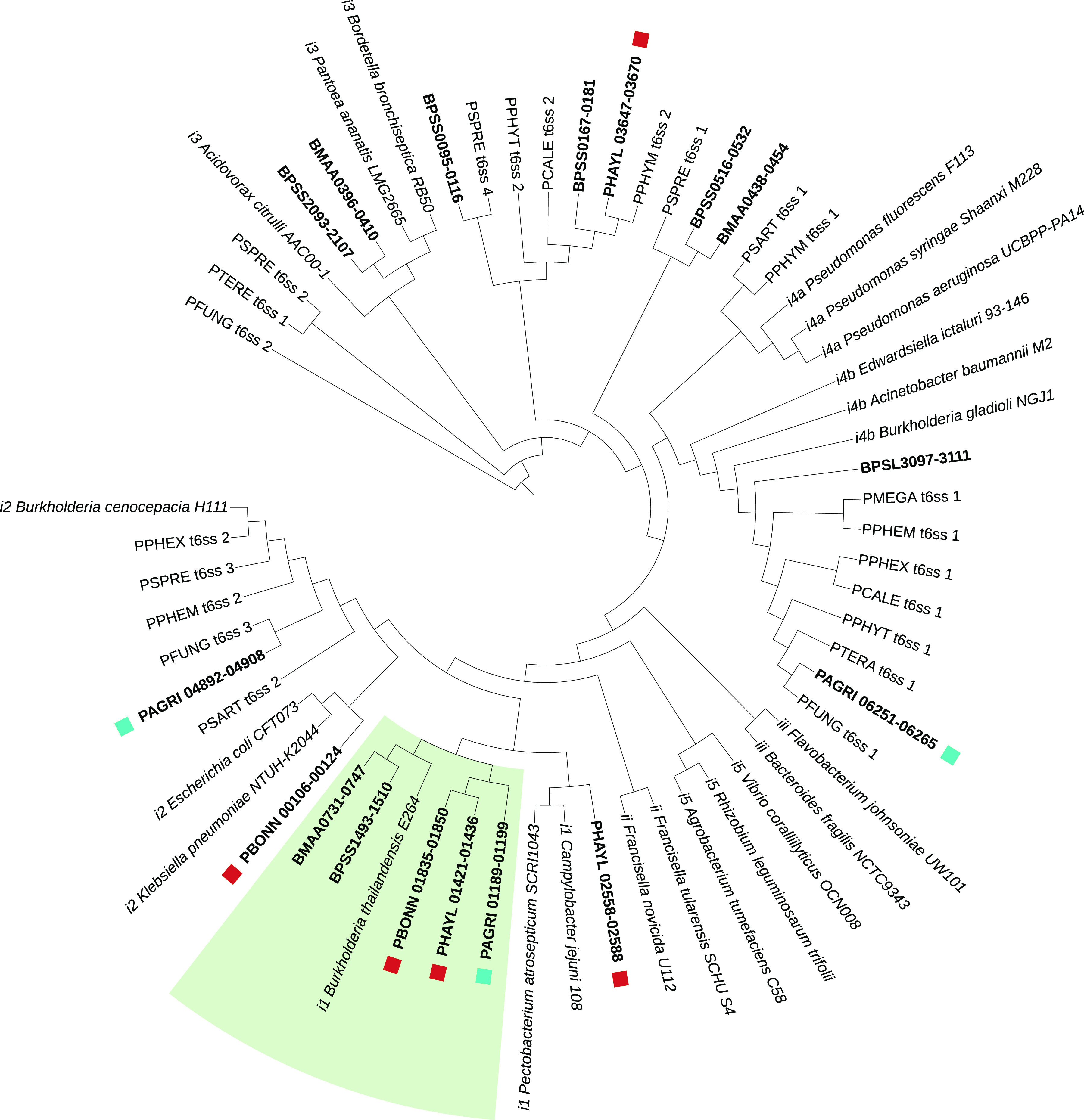
Type 6 secretion systems categorized using the conserved component genes tssB (sheath; COG3516), tssC (sheath; COG3517), and tssF (baseplate; COG3519). Branch lengths were ignored to improve readability of the ASTRAL tree topology. T6SS categories precede the name of the strain to which the operon belongs (e.g., “*ii* Francisella novicida *U112*” belongs to category ii), downloaded from SecReT6 database v3.0 (Li et al. [139]). Tip labels for T6SS in the three D. discoideum-symbiont genomes, B. mallei (BMAA), or B. pseudomallei (BPSS, BPSL) are shown in bold font face with gene IDs for ease of cross-reference. D. discoideum-symbiont genome T3SS operons are marked with a square symbol, and the clade containing the shared T6SS operon is shaded.

Bacterial secretion systems are frequently implicated in host-symbiont interactions (58, 59). All D. discoideum-symbiont genomes possessed multiple type III secretions systems (T3SS) and type VI secretion systems (T6SS) in larger numbers than several of the other *Paraburkholderia* genomes examined (Fig. 5; Table S4). Classification of T3SS showed that one specific T3SS operon shared among D. discoideum symbionts falls into category 8 T3SS (Fig. 6 and Fig. S4). This category of T3SS also includes BurBor found in the plant pathogen *Robbsia* (previously *Burkholderia*) *andropogonis* (60), as well as *Bordatella* species that include mammalian pathogens (61). In addition, one specific T6SS operon is shared among D. discoideum-symbiont genomes and belongs to category i1 (Fig. 7 and Fig. S5). More importantly, this T6SS operon clusters together with the virulence-causing T6SS-5 operon found in Burkholderia mallei, B. pseudomallei, and B. thailandensis (62). B. mallei causes glanders disease and is an obligate pathogen that evolved from an ancestor shared with melioidosis-causing soil bacterium B. pseudomallei (37, 63, 64). B. thailandensis is sister species to the other two and is a facultative pathogen similar to B. pseudomallei but with much lower clinical virulence (62).

10.1128/msystems.00562-22.4 TABLE S4 Secretion systems detected for each *Paraburkholderia* genome examined in this study. Download Table S4, DOCX file, 0.02 MB. Copyright © 2022 Noh et al. 2022 Noh et al. https://creativecommons.org/licenses/by/4.0/ This content is distributed under the terms of the Creative Commons Attribution 4.0 International license.

10.1128/msystems.00562-22.9 FIG S4 Type 3 secretion systems and flagella categorized using gene trees of the conserved component genes sctJ (inner membrane ring; IPR003282), sctN (ATPase; IPR005714), and sctV (export apparatus; IPR006302). Branch lengths were ignored to improve readability of the ASTRID tree topology. T3SS categories precede the name of the operon (e.g., “8 Pand7” is operon Pand7 belonging to category 8), downloaded from T3Enc database v1.0 (135). Tip labels for T3SS in the three D. discoideum-symbiont genomes, B. mallei (BMAA), or B. pseudomallei (BPSS) are shown in bold font face with gene IDs for ease of cross-reference. D. discoideum-symbiont genome T3SS operons are marked with a square symbol, and the clade containing the shared T3SS operon is shaded. Download FIG S4, EPS file, 2.0 MB. Copyright © 2022 Noh et al. 2022 Noh et al. https://creativecommons.org/licenses/by/4.0/ This content is distributed under the terms of the Creative Commons Attribution 4.0 International license.

10.1128/msystems.00562-22.10 FIG S5 Type 6 secretion systems categorized using the conserved component genes tssB (sheath; COG3516), tssC (sheath; COG3517), and tssF (baseplate; COG3519). Branch lengths were ignored to improve readability of the ASTRID tree topology. T6SS categories precede the name of the strain to which the operon belongs (e.g., “*ii* Francisella novicida *U112*” belongs to category ii), downloaded from SecReT6 databse v3.0 (139). Tip labels for the T6SS in the three D. discoideum-symbiont genomes, B. mallei (BMAA), or B. pseudomallei (BPSS, BPSL) are shown in bold font face with gene IDs for ease of cross-reference. D. discoideum-symbiont genome T3SS operons are marked with a square symbol, and the clade containing the shared T6SS operon is shaded. Download FIG S5, EPS file, 2.6 MB. Copyright © 2022 Noh et al. 2022 Noh et al. https://creativecommons.org/licenses/by/4.0/ This content is distributed under the terms of the Creative Commons Attribution 4.0 International license.

The T6SS-5-like and BurBor-like T3SS operons shared by the D. discoideum symbionts are found directly next to each other on the respective genomes of *P. agricolaris*, *P. bonniea*, and *P. hayleyella*. B. pseudomallei and B. thailandensis also have a T3SS (T3SS-3 in the literature) adjacent to their T6SS-5, but these T3SS-3 operons appear to be unrelated to the BurBor-like T3SS found in the D. discoideum-symbiont genomes. It is worth noting that the two adjacent T3SS-3 and T6SS-5 operons in B. pseudomallei and B. thailandensis have been shown to be functionally linked and necessary for virulence, with the T3SS-3 effectors regulating the expression of the adjacent T6SS-5 (42, 65–67).

We attempted to identify effector proteins that might be functionally linked to these D. discoideum-symbiont secretion systems (Table S5). We identified homologs of the T6SS effector VgrG-5 that would likely be associated with the shared T6SS-5-like operon (Table S5). Unexpectedly, VgrG-5 in *P. agricolaris* (gene ID PAGRI_01155) is a homolog but not an ortholog to VgrG-5 in the two reduced genomes (PBONN_01842 and PHAYL_01429). This suggests the possibility of two independent evolutionary origins of this T6SS effector and potentially different functional roles. In Burkholderia thailandensis, VgrG-5 is necessary for postinfection cell-to-cell spread within mammalian hosts (68). For each genome, we predicted additional secretion system effectors, including a chaperonin ClpB and a sodium/solute symporter for *P. agricolaris* and RHS (rearrangement hot spot) proteins that may mediate contact-dependent growth inhibition during bacterial competition for *P. hayleyella*. Finally, we predicted secreted effectors containing eukaryotic domains specific to our Pfam clans of interest (Ank, TPR, LRR, Pentapeptide, F-box, and RING). Previous investigations of amoeba symbiont genomes have observed enrichment of proteins possessing these domains that hypothetically mediate physiological interactions with a eukaryotic host (69–71). Notably, two proteins each directly adjacent to VgrG-5 in *P. agricolaris* (PAGRI_01156-7) and *P. bonniea* (PBONN_01840-1) each contained pentapeptide repeat domains. InterProScan searches indicated that two proteins in *P. hayleyella* (PHAYL_01430-1) adjacent to VgrG-5 also contain pentapeptide repeat domains. However, no known functions are predicted for these protein pairs.

10.1128/msystems.00562-22.5 TABLE S5 Secreted effectors predicted in D. discoideum-symbiont *Paraburkholderia* genomes. Download Table S5, DOCX file, 0.02 MB. Copyright © 2022 Noh et al. 2022 Noh et al. https://creativecommons.org/licenses/by/4.0/ This content is distributed under the terms of the Creative Commons Attribution 4.0 International license.

## DISCUSSION

The genomes of *Paraburkholderia* symbionts of D. discoideum present a unique opportunity to compare the significantly differently sized genomes of three symbiont species that share the ability to persistently infect D. discoideum. We found evidence that relative to the other *Paraburkholderia* genomes we investigated, D. discoideum-symbiont genomes have increased secretion system and motility genes that potentially mediate interactions with their host. Specifically, adjacent type 3 and type 6 secretion system operons shared across all three D. discoideum-symbiont genomes may have an important role. The BurBor-like T3SS operon is closely related to one found in the plant pathogen Robbsia andropogonis. It includes a needle apparatus uncommon among *Burkholderia* T3SS that is used to inject rhizobitoxine into a wide range of plant hosts (60, 72). The adjacent T6SS operon is closely related to T6SS-5 shared by B. mallei, B. pseudomallei, and B. thailandensis. T6SS-5 is functionally important for the intercellular life cycle of these pathogenic *Burkholderia* (68). We hypothesize that the BurBor-like T3SS operon is used during initial host infection and the T6SS-5-like operon may have a functional role postinfection. We also found orthologs to the T6 effector VgrG-5 specific to T6SS-5, as well as two neighboring potential effectors with eukaryote-like pentapeptide repeat domains in the three D. discoideum-symbiont genomes.

Some but not all of the component genes of the shared T6SS-5-like and BurBor-like T3SS operons are among the 120 D. discoideum-symbiont-shared genes not found in any of the other *Paraburkholderia* genomes we compared. It is possible that an unsampled *Paraburkholderia* genome is the source of these secretion systems and other potential symbiosis-mediating factors, including the *bhuRSTUV* iron acquisition genes. However, given the estimated large phylogenetic distance between *P. agricolaris* versus *P. bonniea* and *P. hayleyella*, we hypothesize that one of these clades horizontally acquired the ability to symbiotically associate with D. discoideum from the other. Key genes could have been horizontally transferred among symbiont genomes within the D. discoideum amoeba host environment, especially since different D. discoideum-symbiont species have been found coinfecting amoeba hosts (9). The potential role of protists and amoebas in particular for enabling gene exchange among potential pathogens has been noted before (73, 74) and evidence to support this role continues to accumulate. For example, diverse *Acanthamoeba* symbionts appear to share genes with each other that are functionally enriched for host interaction (75), and the genomes of Mycobacterium that can coinfect amoebas contain several genes with significant homology to other amoeba-resistant bacterial genomes (76). Finally, protist-associated *Parachlamydiaceae* have open pan-genomes that suggest the horizontal acquisition of genes to adapt to different intracellular niches (77). This is in contrast to more specialized obligate intracellular pathogens in the Chlamydiaceae.

While the secretion system features shared among *Paraburkholderia* symbionts of D. discoideum are striking, *P. agricolaris* is otherwise difficult to distinguish from other *Paraburkholderia* based on its genome size and content. However, the reduced genomes of *P. bonniea* and *P. hayleyella* display characteristics indicative of their evolution in a host environment. All three species retain the ability to live outside D. discoideum, but the genomes of *P. bonniea* and *P. hayleyella* show fewer transcriptional regulators, as well as fewer carbohydrate and inorganic ion transporters. The reduced genomes possess a combination of genes with molecular evolution patterns that indicate specific responses to the host environment (both stronger and weaker evolutionary constraints) rather than uniform deterioration under genetic drift. In addition, the lack of IS element proliferation and absence of excessive pseudogene accumulation compared to other *Paraburkholderia* genomes suggest that these already reduced genomes are relatively stable.

These combined pieces of evidence support the view that the reduced-genome D. discoideum symbionts are “professional symbionts,” symbiont lineages that may be ancestrally adapted and possess reduced genomes that are compact and streamlined rather than haphazardly deteriorated (11). Similar to what we observed in the genomes of *P. bonniea* and *P. hayleyella*, streamlined genomes of professional symbionts are expected to have intact core essential genes, including DNA recombination and repair pathways, and various systems that can mediate interactions with their hosts, including secretion systems and effectors (11). We note that genome streamlining as a theory refers to primarily adaptive genome reduction in free-living microbes that is predicted to be the result of a large effective population size and selection that favors lower genome complexity (78, 79). The streamlined rather than deteriorated genomes of *P. bonniea* and *P. hayleyella* do exhibit characteristics that overlap the predicted outcomes of genome streamlining (e.g., intact core metabolic repertoire, lack of pseudogenes and mobile elements, reduced regulatory factors). However, the underlying cause of genome reduction must be distinct given the facultative nature of D. discoideum*-Paraburkholderia* symbiosis. *P. bonniea* and *P. hayleyella* genomes may have evolved under potentially fluctuating regimes of effective population size and selection pressures depending on whether the symbionts were within an amoeba host versus free living in the soil environment. Intraspecific genetic variation appears to be larger for *P. agricolaris* compared to *P. bonniea* or *P. hayleyella* (9), suggesting that *P. agricolaris* host adaptation may be ongoing and more dynamic. We look forward to expanding this work to a larger collection of D. discoideum-symbiont genomes in the future to identify both convergent and divergent host adaptation patterns among D. discoideum symbionts and to continue to add to a growing body of work across diverse protist-prokaryote symbioses.

*Burkholderia sensu lato* include a wide range of species that interact with eukaryotic hosts in symbiotic relationships (80, 81). Our specific results have pointed us mostly toward *Burkholderia* that are mammalian pathogens for clues regarding how D. discoideum and their symbiotic *Paraburkholderia* interact. However, many plants, fungi, and particularly insects have close associations with *Burkholderia* (82). With these other eukaryotic hosts, *Burkholderia* symbionts can offer benefits as diverse as nitrogen fixation or metabolism, pesticide or plant secondary compound degradation, or provide bioactive secondary metabolites as defensive compounds (82). We anticipate drawing upon the rich body of work on *Burkholderia* symbioses in follow-up investigations in the D. discoideum*-Paraburkholderia* system. For example, Burkholderia gladioli strains include pathogens of several plants and defensive symbionts of herbivorous *Lagriinae* beetles (83). Two *B. gladioli* strains isolated from Lagria villosa beetles include a reduced-genome strain that is the dominant symbiont and a nonreduced strain that occurs more sporadically (84). Both strains produce different antifungals that protect L. villosa beetle eggs but can also infect plants and reduce seed production (83, 85, 86). In contrast to *P. bonniea* and *P. hayleyella*, the reduced-genome *B. gladioli* strain is missing genes for several metabolic pathways and DNA repair. The horizontal gain of genes for synthesis of the defensive compound lagriamide is thought to be the key event that led to symbiosis establishment between L. villosa beetles and a plant-associated *B. gladioli* ancestor of the reduced-genome strain (84).

### Conclusion.

Among the three *Paraburkholderia* symbionts of D. discoideum, the genome size and content of *P. agricolaris* are similar to other *Paraburkholderia* while the reduced genomes of *P. bonniea* and *P. hayleyella* display characteristics of genome streamlining rather than deterioration during host adaptation. Despite these differences, we found adjacent type 3 and type 6 secretion system operons shared across all three D. discoideum-symbiont genomes that may have an important role in host-symbiont interactions. These and other shared features suggest a role for horizontal gene transfer within the amoeba host environment that may contribute to the unique ability of these symbionts to establish and maintain a symbiotic relationship with D. discoideum.

## MATERIALS AND METHODS

### *Paraburkholderia* genome selection and gene prediction.

Genome sequencing methods were described previously (10). Briefly, we prepared high-quality DNA from individual strains grown on SM/5 agar media using Qiagen Genomic tips (20/G). Two genomes (*P. agricolaris* and *P. bonniea*) were sequenced by the University of Washington PacBio Sequencing Services, and *P. hayleyella* was sequenced by the Duke University Center for Genomic and Computational Biology, all on the PacBio SMRT II platform. Reads were assembled via HGAP versions 1.87 and 1.85 (87). After an initial round of annotation, we identified the chromosomal replication initiator *dnaA* sequence and *initiator replication protein* in each assembly's contig and reoriented each contig from these genes using Circlator (88). We used SMRT analysis software Quiver to repolish each assembly (87).

We chose the following *Paraburkholderia* with finished genomes for more detailed comparison: *P. fungorum* strain ATCC BAA-463 (89), originally isolated from the fungus Phanerochaete chrysosporium (90); *P. sprentiae* strain WSM5005 (91) isolated from root nodules of the domesticated legume *Lebeckia ambigua*; *P. terrae* strain DSM17804 (92) isolated from broad-leaved forest soil; and *P. xenovorans* strain LB400 (93) isolated from polychlorinated biphenyl-contaminated soil. We refer to these four as our representative *Paraburkholderia* genomes. For broader scale analyses of molecular evolution and comparative genomics, we used eight additional *Paraburkholderia* genomes that span the clade that includes *P. agricolaris*, *P. hayleyella*, and *P. bonniea*. We added four plant-associated species genomes (*P. megapolitana* LMG23650, *P. phenoliruptrix* BR3459a, *P. phymatum* STM815, *P. phytofirmans* PsJN) and four free-living species genomes (*P. caledonica* PHRS4, *P. phenazinium* LMG2247, *P. sartisoli* LMG24000, and *P. terricola* mHS1) (94–100). All genomes were downloaded from NCBI and considered complete (Table S1). While the genomes of *P. sartisoli* and *P. phenazinium* are fragmented into multiple contigs, all remaining selected genomes are finished into full-length chromosomes (101).

10.1128/msystems.00562-22.1 TABLE S1 *Paraburkholderia* genomes examined in this study (including RefSeq assembly accessions). Download Table S1, DOCX file, 0.02 MB. Copyright © 2022 Noh et al. 2022 Noh et al. https://creativecommons.org/licenses/by/4.0/ This content is distributed under the terms of the Creative Commons Attribution 4.0 International license.

We reannotated each genome with Prokka v1.14.6 (102) using the annotation file of Burkholderia pseudomallei strain K96243 (downloaded from Burkholderia Genome DB version 9.1) as a source of known proteins. Next, we found putative pseudogenes in each genome using Pseudofinder v1.0 (103) with DIAMOND v2.0.6.144 (104) BLAST against the NCBI RefSeq nonredundant protein database (downloaded August 27, 2021) in Annotate mode. Genes predicted to be pseudogenes due to truncation (less than 65% of average length of similar genes by default) or fragmentation (adjacent predicted reading frames match the same known protein) were removed from further analysis.

### Whole-genome alignment.

The genome aligner progressiveMauve (105) identifies locally colinear blocks (LCBs), local alignments that occur in the same sequence order and orientation across multiple genomes. We used all 13 finished *Paraburkholderia* genomes (all genomes noted above except *P. sartisoli* and *P. phenazinium*) for the initial whole-genome progressiveMauve alignment in Mauve v2015-02-25. Next we compared the positions and orientations of locally colinear blocks across our three D. discoideum-symbiont genomes and each of these against the four representative *Paraburkholderia* genomes to identify large-scale synteny using hive plots (106). We used ggraph v2.0.3 and igraph v1.2.11 in R v3.6.0 (107) to generate the hive plots.

### Horizontally transferred genetic element detection.

We used the ISFinder (108) webserver (accessed 17 October 2021) and its nucleotide BLAST to identify putative IS elements to test for their proliferation in each genome. We identified the best hits by comparing overlapping hits by E value and bit score. We retained hits that were at least 70% coverage of the IS element it matched in the database.

Genomic islands are clusters of genes of horizontally transferred origin and have been found in a range of sizes from as small as 5 to as large as 500 kb (109–111). We applied IslandPath-DIMOB and SIGI-HMM as implemented via the IslandViewer 4 webserver (112). IslandPath-DIMOB uses sequence composition and mobility genes, while SIGI-HMM uses codon usage bias. We then used pairwise reciprocal megablast to determine whether any of the predicted genomic islands were shared among D. discoideum-symbiont genomes.

Finally, we looked for individually occurring horizontally transferred genes using DarkHorse2 v2.0_rev09. DarkHorse2 compares individual genes against the NCBI NR database and detects genes with unusual distributions of hits by calculating a lineage probability index (LPI) score (113, 114). Vertically inherited genes will have a high-LPI score because most high-scoring BLASTP hits will belong to close taxonomic relatives. Horizontally transferred genes are detected because high-scoring BLASTP hits will be taxonomically distant, leading to lower LPI scores. We used DIAMOND to perform BLASTP, and then following suggestions from the author, excluded self and sister species hits (*P. fungorum* for *P. agricolaris*, *P. bonniea*, and *P. hayleyella* from each other) and set the global filter threshold to 0.02 to allow candidate matches to have bit scores up to 2% different from the best nonself match.

### Gene functional annotation.

We performed broad-scale functional annotation with Clusters of Orthologous Groups (COG) (115, 116) and Kyoto Encyclopedia of Genes and Genomes (KEGG) Orthology (KO) (117, 118). We assigned COG by RPS-BLAST (119) against COG position-specific scoring matrices downloaded from the NCBI Conserved Domain Database (version 31 July 2019). We followed JGI MGAP v4 practices and used an E-value cutoff of 0.01 and query coverage of at least 70% to be considered a valid assignment (120). We assigned KO using the BlastKOALA webserver (http://kegg.jp/blastkoala/; accessed 13 to 28 July 2020) that performed BLASTP against the KEGG GENES database at the prokaryote Genus and eukaryote Family level (121).

We compared functional genome composition in terms of the numbers of genes observed in each COG category using agglomerative clustering and nonmetric multidimensional scaling (NMDS). Both were implemented in R: agglomerative clustering using cluster v2.1.2, and NMDS using vegan v2.5-7. In both analyses, the reduced genomes of *P. bonniea* and *P. hayleyella* comprise their own cluster while all other genomes clustered together. We compared genome statistics by cluster, including genome size, GC% (proportion of GC nucleotides in the genome), and proportions of intact genes versus pseudogenes. To determine which COG categories contribute to this difference, we used a binomial exactTest (122) using edgeR v3.26.8 (123). Because the enrichment of functional categories of genes for the comparison of *P. bonniea* and *P. hayleyella* versus other *Paraburkholderia* genomes may be due to the maintenance of necessary genes despite genome size degradation, we looked at both normalized and raw count comparisons. For each COG category that was significantly differently detected between the two clusters both in the relative (postnormalization) and absolute (raw counts) sense, we investigated which specific COGs were contributing to the difference. We used KEGG Mapper (124) and its Reconstruct Pathway tool (https://www.genome.jp/kegg/tool/map_pathway.html; accessed 14 March 2022) to corroborate differences in pathway components in genomes based on KO annotations.

### Core genome molecular evolution.

To determine orthologous genes shared among all examined genomes, we performed a pan-genome analysis using Roary v3.13.0 (125) with a 70% identity threshold. To test hypotheses regarding changes in lineage-specific rates of molecular evolution in *Paraburkholderia* symbionts of D. discoideum, we used core genes detected in the Roary pan genome analysis. We used the whole-genome species tree from Brock et al. (10) and dropped any additional taxa using the drop.tip() function in phytools v1.0-1 in R. This species tree was used throughout the subsequent molecular evolution analyses using PAML v4.9d (126). Protein sequence multifasta files for each core gene were aligned with MUSCLE v3.8.31 (127) and then converted into codon alignments using PAL2NAL v14 (128). We ran a series of codeml analyses on each codon alignment with proportional branch lengths as recommended by the PAML manual.

We applied three alternative hypotheses that test whether patterns of molecular evolution were altered by a symbiotic lifestyle (“symbiotic”), association specifically with D. discoideum (“dicty”), or in the reduced genomes of *P. bonniea* and *P. hayleyella* (“reduced”) (Table S1). We compared each of their Akaike Information Criterion (AIC) scores to that of the null hypothesis (H0) that there should be no significant variation in molecular evolution across the species tree. AIC attempts to minimize information loss in order to select the “best” model (129, 130). The hypothesis with the smallest AIC score with at least a 1-point difference from the null hypothesis was considered the best fit. For genes that showed patterns of molecular evolution that best fit an alternative hypothesis, we used Wilcoxon signed-rank tests in R to compare estimates of dN/dS, the ratio of nonsynonymous substitutions per nonsynonymous site over synonymous substitutions per synonymous site, between groups of species.

### Essential amino acid biosynthetic repertoire.

We used GapMind webserver (http://papers.genomics.lbl.gov/cgi-bin/gapView.cgi; accessed 26 January 2022) to evaluate any loss of essential amino acid biosynthesis pathways in each genome. GapMind detects genes involved in the biosynthesis of 17 amino acids (all standard amino acids excluding alanine, aspartate, and glutamate) and chorismate based on MetaCyc pathways using a combination of sequence similarity and protein family profiles (131, 132). It can handle fusion proteins (two enzymes fused into a single protein) and split proteins (multidomain enzyme split into up to two proteins).

### Protein secretion system repertoire and effector prediction.

We used TXSScan (133) implemented in Galaxy/Pasteur (accessed 2 October 2020) to identify protein secretion systems in the three focal and 12 additional *Paraburkholderia* genomes. TXSScan identifies protein secretion systems (types I to VI and IX, including type IV and tight adherence [Tad] pili) and flagella based on 204 experimentally studied protein profiles. It also determines whether a secretion system is complete by the presence of mandatory and forbidden component genes by subtype, and whether it is contained within a single operon (single locus) or across a few neighboring operons (multilocus). We used the genomes of Burkholderia mallei ATCC 23344 and Burkholderia pseudomallei K96243 here to serve as ground truth because their secretion systems are well studied. A small number of T6SS and one T3SS were classified as incomplete due to misidentifying secretion system component homologs (e.g., TssC as IglB). These were manually corrected and included in the analyses. We verified these manually corrected operons against secretion system databases and Burkholderia Genome DB v9.1 (134).

We classified all T3SS and T6SS found in our 15 genomes. For T3SS, we used the T3Enc database v1.0 (135) and downloaded three representative amino acid sequences of 13 categories of T3SS for the conserved component genes sctJ (inner membrane ring; IPR003282), sctN (ATPase; IPR005714), and sctV (export apparatus; IPR006302). We aligned protein sequences of each component gene using MUSCLE v3.8.31 (127) and made gene trees using the Le and Gascuel substitution model with FastTree v2.1.10 (136). We estimated a species tree from these gene trees using ASTRID v2.2.1 (137) and ASTRAL v5.7.8 (138). We followed the same methods for T6SS using the SecReT6 database v3.0 (139) and the conserved component genes tssB (sheath; COG3516), tssC (sheath; COG3517), and tssF (baseplate; COG3519).

We used VFDB (Virulence factors of Pathogenic Bacteria; accessed 25 January 2022) (140) and downloaded protein sequences of known *Bordatella* T3 Secreted Effectors and *Burkholderia* T3 and T6 Secreted Effectors. We used DIAMOND BLASTP and these proteins as query sequences against the predicted amino acid sequences of each genome. We also used the webserver BastionHub (accessed 20 April 2021) to predict secreted effectors. BastionHub (141) combines a hidden Markov model-based approach and a machine learning approach. Finally, we used effectiveELD (142, 143) on the effectiveDB server (accessed 28 March 2022) to find putative secreted proteins that contain a eukaryotic-like domain. We specifically looked for proteins with domains that belong to Pfam clans for Ank (ankyrin), TPR (tetratricopeptide repeat), LRR (leucine-rich repeat), Pentapeptide, F-box, and RING (including U-box). These domains were selected based on previous reports regarding large numbers of proteins containing eukaryotic domains among amoeba symbionts (69–71). InterProScan (144) webserver (accessed 29 March 2022) was used for additional investigation of secreted effector candidates.

### *Paraburkholderia* genome browser.

We built a web Genome Browser for each D. discoideum-symbiont genome for convenient browsing of all annotated genomic features mentioned above. We used JBrowse v1 (145, 146). The front-end web application was developed in Centos Steam 8 version of Linux. We used NGINX Web Server v1.14.1 and Java OpenJDK v1.8.0_322. The browser is available at https://burk.colby.edu. The GitHub repositories supporting the browser are available at https://github.com/noh-lab/burk-browser and https://github.com/noh-lab/jbrowse-executables.

### Data and code availability.

All analyses and figures found in this article can be generated and recreated using input data and code available at the GitHub repository https://github.com/noh-lab/comparative-dicty-symbionts.
