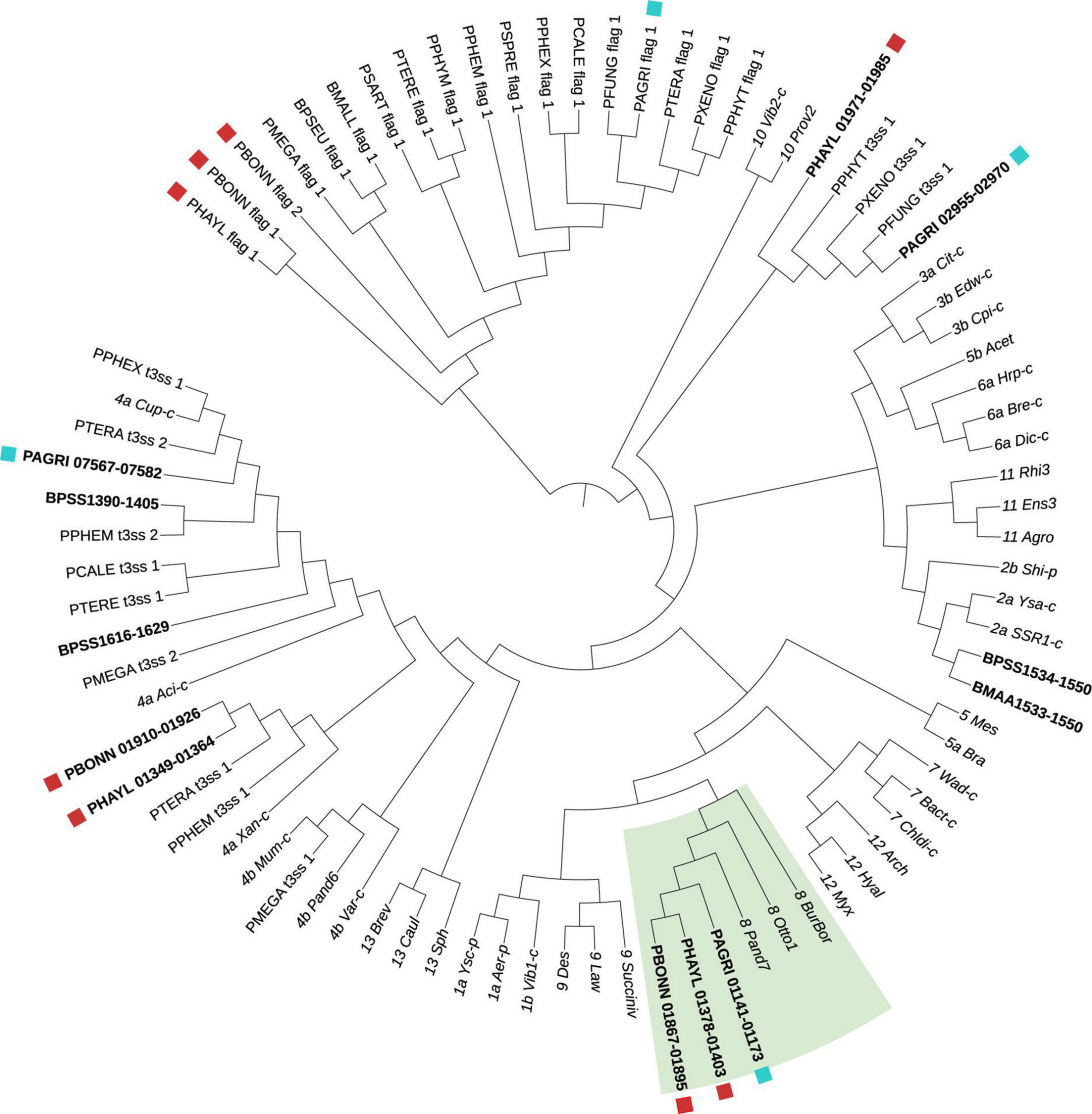# Correction for Noh et al., “Reduced and Nonreduced Genomes in *Paraburkholderia* Symbionts of Social Amoebas”

**DOI:** 10.1128/msystems.01065-24

**Published:** 2024-09-18

**Authors:** Suegene Noh, Benjamin J. Capodanno, Songtao Xu, Marisa C. Hamilton, Joan E. Strassmann, David C. Queller

## AUTHOR CORRECTION

Volume 7, no. 5, e00562-22, 2022, https://doi.org/10.1128/msystems.00562-22. Page 10, Fig. 6: Two *P. bonniea* branch tip labels include errors in their gene IDs. The figure should appear as shown in this author correction. These corrections do not affect the results and the conclusion.

Supplemental material, Fig. S4: Two *P. bonniea* branch tip labels include errors in their gene IDs. The correct figure is given in the revised supplemental file in this author correction. These corrections do not affect the results and the conclusion.

We apologize for any inconvenience caused by these errors.

**Fig 6:**