# Supplementary material for: Reduced and Nonreduced Genomes in Paraburkholderia Symbionts of Social Amoebas
Source: mSystems. 2022 Sep 13;7(5):e00562-22. doi: 10.1128/msystems.00562-22 (PMC9601139; doi:10.1128/msystems.00562-22)
Supplement: TABLE S2 [file msystems.00562-22-s0007.docx]

Table S2. Insertion Sequence (IS) elements found in *D. discoideum*-symbiont *Paraburkholderia* genomes

| Genome | IS1090  (IS256 family) | ISBmu21  (IS6 family) | ISBp1  (IS3 family) | ISBuph1  (IS5 family) | ISPa37  (IS30 family) |
| --- | --- | --- | --- | --- | --- |
| *P. agricolaris* BaQS159 | 6 | 1 | 0 | 0 | 0 |
| *P. bonniea* BbQS859 | 0 | 0 | 2 | 3 | 0 |
| *P. hayleyella* BhQS11 | 0 | 0 | 0 | 0 | 1 |
