# Supplementary material for: Reduced and Nonreduced Genomes in Paraburkholderia Symbionts of Social Amoebas
Source: mSystems. 2022 Sep 13;7(5):e00562-22. doi: 10.1128/msystems.00562-22 (PMC9601139; doi:10.1128/msystems.00562-22)
Supplement: TABLE S4 [file msystems.00562-22-s0009.docx]

Table S4. Secretion systems detected for each *Paraburkholderia* genome examined in this study

| genome | T1SS | T2SS | Tad | T3SS | Flagella | pT4SS | cT4SS | T5aSS | T5bSS | T5cSS | T6SSi |
| --- | --- | --- | --- | --- | --- | --- | --- | --- | --- | --- | --- |
| PAGRI | 0 | 1 | 1 | 3 | 1 | 0 | 1 | 2 | 2 | 3 | 3 |
| PBONN | 3 | 1 | 1 | 2 | 2 | 0 | 0 | 3 | 3 | 3 | 2 |
| PHAYL | 2 | 1 | 1 | 3 | 1 | 0 | 0 | 3 | 3 | 5 | 3 |
| PCALE | 2 | 1 | 0 | 1 | 1 | 0 | 0 | 0 | 2 | 3 | 2 |
| PFUNG | 1 | 1 | 2 | 1 | 1 | 0 | 0 | 2 | 5 | 2 | 3 |
| PMEGA | 4 | 1 | 1 | 1 | 1 | 0 | 0 | 0 | 6 | 3 | 1 |
| PPHEM | 3 | 1 | 2 | 2 | 1 | 0 | 0 | 1 | 1 | 5 | 2 |
| PPHEX | 2 | 1 | 2 | 1 | 1 | 1 | 2 | 0 | 7 | 4 | 2 |
| PPHYM | 2 | 1 | 1 | 0 | 1 | 0 | 1 | 0 | 5 | 5 | 2 |
| PPHYT | 1 | 1 | 2 | 1 | 1 | 0 | 3 | 1 | 2 | 3 | 2 |
| PSART | 2 | 1 | 1 | 0 | 1 | 0 | 1 | 3 | 3 | 1 | 2 |
| PSPRE | 2 | 1 | 0 | 0 | 1 | 1 | 1 | 1 | 6 | 3 | 4 |
| PTERA | 2 | 1 | 2 | 2 | 1 | 0 | 0 | 0 | 2 | 4 | 1 |
| PTERE | 2 | 1 | 1 | 1 | 1 | 1 | 0 | 0 | 5 | 3 | 1 |
| PXENO | 3 | 1 | 1 | 1 | 1 | 0 | 4 | 0 | 5 | 2 | 0 |

* T4SS are classified into protein secretion vs. conjugation-related types (pT4SS vs. cT4SS)
