# Supplementary material for: Reduced and Nonreduced Genomes in Paraburkholderia Symbionts of Social Amoebas
Source: mSystems. 2022 Sep 13;7(5):e00562-22. doi: 10.1128/msystems.00562-22 (PMC9601139; doi:10.1128/msystems.00562-22)
Supplement: TABLE S5 [file msystems.00562-22-s0010.docx]

Table S5. Secreted effectors predicted in *D. discoideum*-symbiont *Paraburkholderia* genomes

| Genome | Gene ID | Annotation | Annotation source |
| --- | --- | --- | --- |
| ***P. agricolaris* BaQS159** | PAGRI_00020 | Pentapeptide domain protein | effectiveELD |
|  | PAGRI_00362 | TPR domain protein | effectiveELD |
|  | PAGRI_00592 | TPR domain protein | effectiveELD |
|  | PAGRI_00623 | TPR domain protein | effectiveELD |
|  | PAGRI_01039 | Pentapeptide domain protein | effectiveELD |
|  | PAGRI_01112 | TPR domain protein | effectiveELD |
|  | PAGRI_01155 | T6SS effector VgrG-5 | VFDB |
|  | PAGRI_01156 | Pentapeptide domain protein | effectiveELD |
|  | PAGRI_01157 | Pentapeptide domain protein | effectiveELD |
|  | PAGRI_01179 | TPR domain protein | effectiveELD |
|  | PAGRI_01202 | LRR domain protein | effectiveELD |
|  | PAGRI_02308 | T6SS effector | BastionHub |
|  | PAGRI_02840 | TPR domain protein | effectiveELD |
|  | PAGRI_02920 | TPR domain protein | effectiveELD |
|  | PAGRI_03740 | TPR domain protein | effectiveELD |
|  | PAGRI_04081 | TPR domain protein | effectiveELD |
|  | PAGRI_04899 | T6SS effector VgrG-5 | VFDB |
|  | PAGRI_04916 | T4SS effector | BastionHub |
|  | PAGRI_05317 | T3SS effector | BastionHub |
|  | PAGRI_06262 | TPR domain protein | effectiveELD |
|  | PAGRI_06513 | TPR domain protein | effectiveELD |
|  | PAGRI_07504 | TPR domain protein | effectiveELD |
| ***P. bonniea* BbQS859** | PBONN_00263 | Ank domain protein | effectiveELD |
|  | PBONN_00282 | T1SS effector | BastionHub |
|  | PBONN_00789 | T3SS effector | BastionHub |
|  | PBONN_01051 | Pentapeptide domain protein | effectiveELD |
|  | PBONN_01823 | T1SS effector | BastionHub |
|  | PBONN_01840 | Pentapeptide domain protein | effectiveELD |
|  | PBONN_01841 | Pentapeptide domain protein | effectiveELD |
|  | PBONN_01842 | T6SS effector VgrG-5 | VFDB |
|  | PBONN_01861 | TPR domain protein | effectiveELD |
|  | PBONN_02315 | T1SS effector | BastionHub |
|  | PBONN_02901 | LRR domain protein | effectiveELD |
|  | PBONN_03205 | Pentapeptide domain protein | effectiveELD |
|  | PBONN_03247 | T6SS effector | BastionHub |
|  | PBONN_03375 | Ank domain protein | effectiveELD |
|  | PBONN_03418 | T1SS effector | BastionHub |
| ***P. hayleyella* BhQS11** | PHAYL_00296 | T1SS effector | BastionHub |
|  | PHAYL_00422 | T6SS effector | BastionHub |
|  | PHAYL_00425 | T6SS effector | BastionHub |
|  | PHAYL_00686 | T1SS effector | BastionHub |
|  | PHAYL_01212 | T6SS effector | BastionHub |
|  | PHAYL_01429 | T6SS effector VgrG-5 | VFDB |
|  | PHAYL_02569 | T6SS effector | BastionHub |
|  | PHAYL_02573 | T6SS effector | BastionHub |
|  | PHAYL_02589 | T6SS effector | BastionHub |
|  | PHAYL_02594 | T6SS effector | BastionHub |
|  | PHAYL_02596 | T6SS effector | BastionHub |
|  | PHAYL_02973 | T2SS effector | BastionHub |
|  | PHAYL_02975 | T6SS effector | BastionHub |
|  | PHAYL_02976 | T6SS_effector | BastionHub |
|  | PHAYL_03032 | Ank domain protein | effectiveELD |
