# Supplementary material for: Reduced and Nonreduced Genomes in Paraburkholderia Symbionts of Social Amoebas
Source: mSystems. 2022 Sep 13;7(5):e00562-22. doi: 10.1128/msystems.00562-22 (PMC9601139; doi:10.1128/msystems.00562-22)
Supplement: TABLE S1 [file msystems.00562-22-s0006.docx]

Table S1. *Paraburkholderia* genomes examined in this study

| Genome (species and strain) | Abbreviation | Category | RefSeq assembly accession |
| --- | --- | --- | --- |
| *P. agricolaris* BaQS159 | PAGRI | *D. discoideum*-symbiont | GCF_009455635.1_ASM945563v1_genomic.fna |
| *P. bonniea* BbQS859 | PBONN | *D. discoideum*-symbiont | GCF_009455625.1_ASM945562v1_genomic.fna |
| *P. hayleyella* BhQS11 | PHAYL | *D. discoideum*-symbiont | GCF_009455685.1_ASM945568v1_genomic.fna |
| *P. fungorum* ATCC BAA-463 | PFUNG | Symbiotic | GCF_000961515.1_ASM96151v1_genomic.fna |
| *P. megapolitana* LMG23650 | PMEGA | Symbiotic | GCF_007556815.1_ASM755681v1_genomic.fna |
| *P. phenoliruptrix* BR3459a | PPHEX | Symbiotic | GCF_000300095.1_ASM30009v1_genomic.fna |
| *P. phymatum* STM815 | PPHYM | Symbiotic | GCF_000020045.1_ASM2004v1_genomic.fna |
| *P. phytofirmans* PsJN | PPHYT | Symbiotic | GCF_000020125.1_ASM2012v1_genomic.fna |
| *P. sprentiae* WSM5005 | PSPRE | Symbiotic | GCF_001865575.1_ASM186557v1_genomic.fna |
| *P. caledonica* PHRS4, | PCALE | Free-living | GCF_003330745.1_ASM333074v1_genomic.fna |
| *P. phenazinium* LMG2247 | PPHEM | Free-living | GCF_900100735.1_IMG2651870170_genomic.fna |
| *P. sartisoli* LMG24000 | PSART | Free-living | GCF_900107685.1_IMG2651870102_genomic.fna |
| *P. terricola* mHS1 | PTERA | Free-living | GCF_003330825.1_ASM333082v1_genomic.fna |
| *P. terrae* DSM17804 | PTERE | Free-living | GCF_002902925.1_ASM290292v1_genomic.fna |
| *P. xenovorans* LB400 | PXENO | Free-living | GCF_000756045.1_ASM75604v1_genomic.fna |
